# Neurosyphilis vasculitis manifesting as ischemic stroke

**DOI:** 10.1590/0037-8682-0546-2019

**Published:** 2020-03-16

**Authors:** Laisson de Moura Feitoza, Raquel Silveira Bello Stucchi, Fabiano Reis

**Affiliations:** 1 Universidade Estadual de Campinas, Faculdade de Ciências Médicas, Departamento de Radiologia, Campinas, SP, Brasil.; 2 Universidade Estadual de Campinas, Faculdade de Ciências Médicas, Departamento de Clínica Médica, Campinas, SP, Brasil.

**FIGURE 1:**
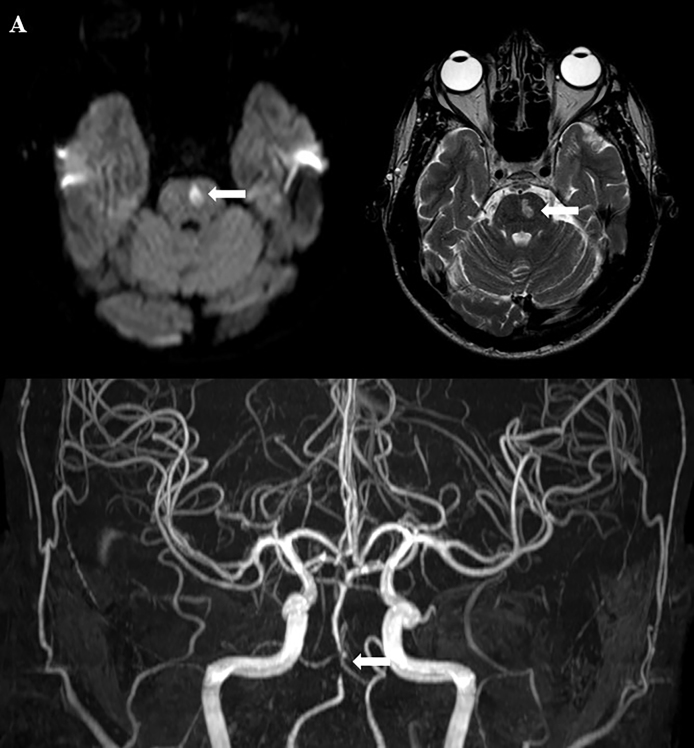
Axial DWI and T2-weighted MRI showing left hemipons infarction (**arrows, A and B)**. Three-dimensional time-of-flight magnetic resonance angiography showing significant stenosis of the basilar artery (**arrow, C**).

**FIGURE 2:**
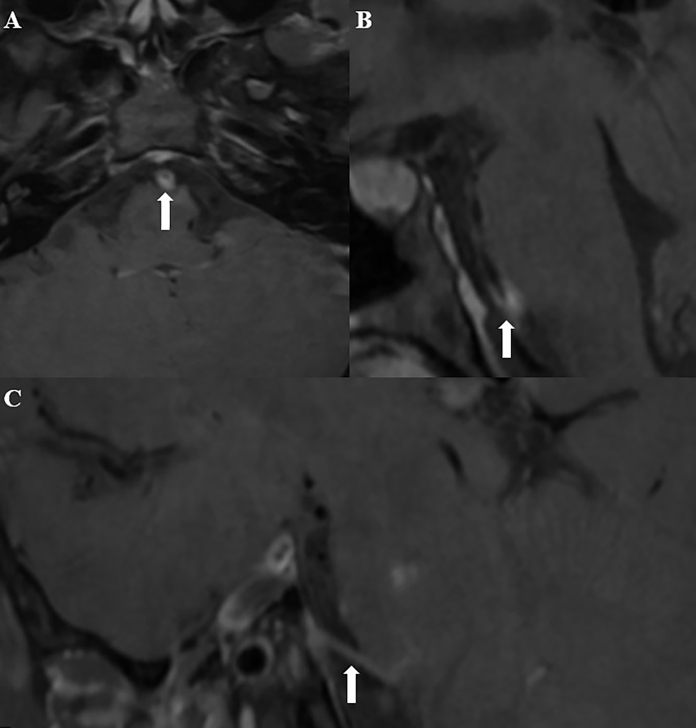
Post-contrast axial and sagittal HR-VWI showing concentric parietal thickening and enhancement of the basilar artery consistent with a vasculitic pattern (**arrows, A and B**). Post-contrast sagittal HR-VWI also reveals enhancement of the left abducens nerve (**arrow, C**).

A 26-year-old man presented with acute-onset right hemiparesis, diplopia on horizontal gaze, and fever. Brain magnetic resonance imaging (MRI) with high-resolution vessel wall imaging (HR-VWI) showed left hemipons infarction and concentric parietal thickening of the basilar artery, consistent with vasculitis (Figures 1 and 2). Cerebrospinal fluid and blood samples were positive for syphilis on the Venereal Disease Research Laboratory (VDRL) test and *Treponema pallidum* particle agglutination assay. A test for human immunodeficiency virus was positive (viral load: 188,330 copies/mL) and his CD4+ count (248 cells/mL) was below the reference range (500-1,450 cells/mL). The patient was administered intravenous penicillin G for 21 days as well as highly active antiretroviral therapy. The serum VDRL test result fell in response to treatment and a VDRL test of his cerebrospinal fluid revealed negative results. The patient was discharged 21 days after admission with residual right hemiparesis and diplopia. 

Neurosyphilis can occur at any stage of the disease and may be associated with occlusive large vessel infarcts^1^ ^-^ ^3^. In such cases, MRI with HR-VWI can be useful for diagnosing stroke and depicting vessel wall inflammation associated with infectious vasculitis.
